# A Case of Acute Heart Failure Following Immunotherapy for Metastatic Lung Cancer

**DOI:** 10.7759/cureus.8093

**Published:** 2020-05-13

**Authors:** Ammar Al-Obaidi, Nathaniel A Parker, Khalil Choucair, Joel Alderson, Jeremy M Deutsch

**Affiliations:** 1 Internal Medicine, University of Kansas School of Medicine, Wichita, USA; 2 Pathology, Ascension Via Christi St. Francis Hospital, Wichita, USA; 3 Hematology / Oncology, University of Kansas School of Medicine, Wichita, USA

**Keywords:** metastatic non-small cell lung cancer, ctla-4 inhibitors, pd-1 inhibitors, cancer immunotherapy, immune-checkpoint inhibitors, autoimmune cardiotoxicity, autoimmune myocarditis, autoimmune heart disease, life threatening

## Abstract

Inhibitors of cytotoxic T-lymphocyte-associated antigen-4, programmed cell death protein-1, and programmed death-ligand 1 have been shown to produce significant antitumor activity in multiple malignancies, and have become essential oncology standard-of-care therapies. Despite their success, the checkpoint inhibitors’ ability to amplify the immune system response against tumor cells has been associated with a unique panel of side effects known as immune-related adverse events. The involvement of the myocardium has been reported previously, but it’s remarkably uncommon. Even more noteworthy is that secondary autoimmune myocarditis and heart failure due to these medications are typically fatal.

## Introduction

Tumor cells have been shown to evade the host’s immune system through various mechanisms including the down-regulation of lymphocytic T-cells via activation of inhibitory checkpoint receptors [1]. Immune checkpoint inhibitors harness the power of the endogenous immune system by blocking these inhibitory interactions between tumor cells and T-cells. Inhibitors of cytotoxic T-lymphocyte-associated antigen-4 (CTLA-4; e.g. ipilimumab) and programmed cell death protein-1 (PD-1; e.g. nivolumab, pembrolizumab, cemiplimab) receptors on T-cells, as well as inhibitors of programmed death-ligand 1 (PD-L1; e.g. durvalumab, avelumab, atezolizumab) on tumor cells have been shown to produce significant anti-tumor activity in multiple malignancies, and have become essential oncology standard-of-care therapies [2].

Despite their success, the checkpoint inhibitors’ ability to amplify the immune system response against tumor cells has been associated with a unique panel of side effects known as immune-related adverse events (irAEs). Since immune checkpoints regulate auto-reactivity, irAEs are thought to reflect auto-immune response mechanisms to checkpoint blockade. Classic irAEs involve the skin (e.g. rash and pruritus), gastrointestinal system (e.g. colitis), endocrine organs (e.g. hypothyroidism and hypophysitis), lungs (e.g. pneumonitis), kidneys (e.g, renal insufficiency), joints (e.g. arthritis) and liver (e.g. hepatitis) [3, 4].

The involvement of the myocardium has been reported previously, but remains an extremely rare adverse event [5]. Of the less than 0.3% of patients who experience acute heart failure and myocarditis due to immune checkpoint inhibitors, the majority develop signs and symptoms of acute heart failure symptoms in the later cycles of immunotherapy [6, 7]. Here, we report a case of autoimmune myocarditis and acute heart failure in female with metastatic non-small cell lung cancer (NSCLC) after treatment with a CTLA-4 plus PD-1 inhibitor. 

## Case presentation

**Figure 1:**
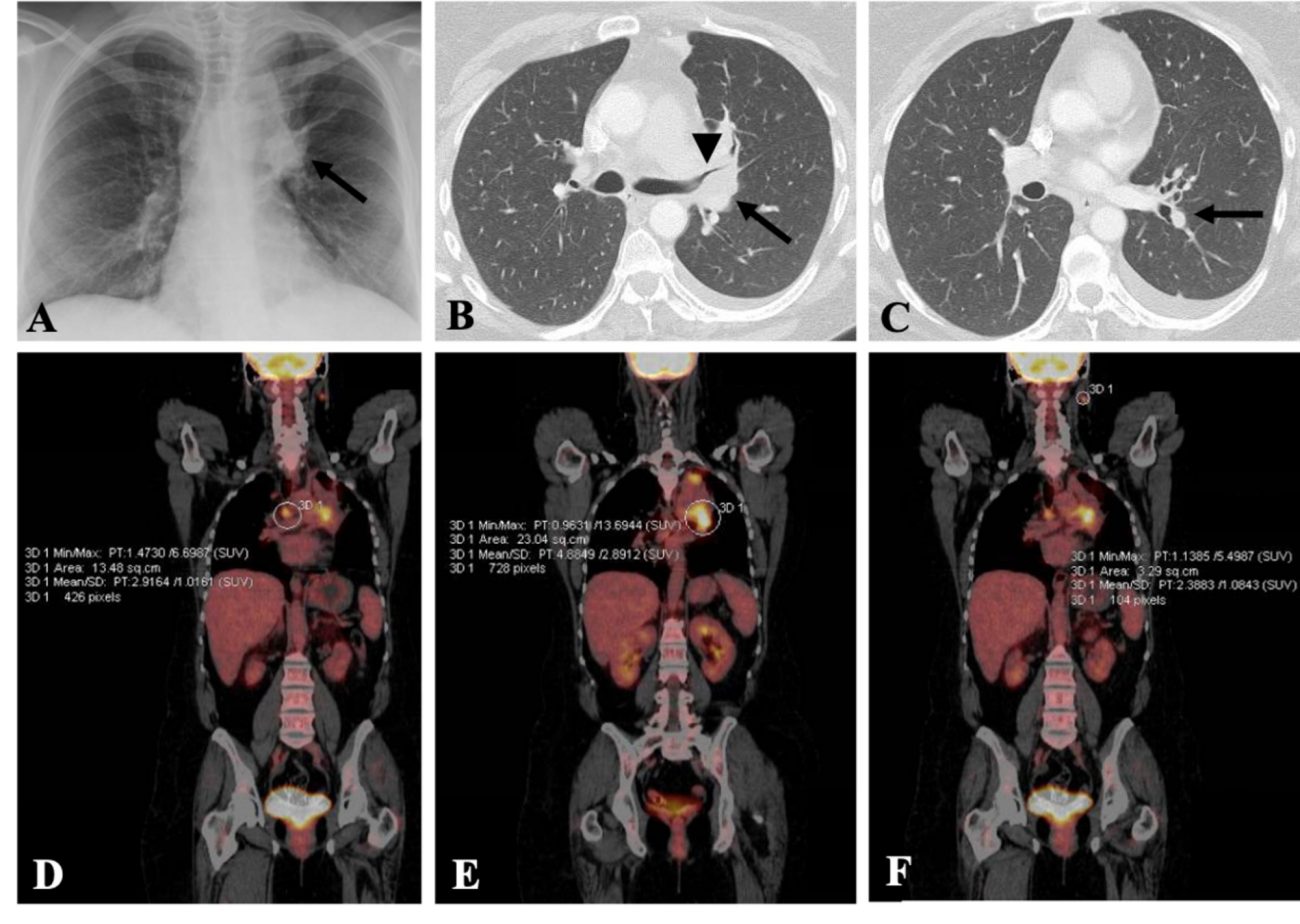
Imaging demonstrates a metastatic lung cancer. (A) Chest x-ray shows a new abnormal rounded density identified in the left hilum. (B) Left upper lobe perihilar mass (*arrow*) measures approximately 3.8 cm, invades the mediastinum, causes complete obstruction of the left upper lobe segmental bronchi (*arrowhead*), and is associated with an enlarged contralateral right upper paratracheal lymph node. (C) Indeterminate 3 mm subpleural nodule in the left lower lobe concerning for metastasis. (D-F) PET-avid disease represented by strong uptake and associated standardized uptake values (SUVs) in the left hilar pulmonary mass, left upper lobe lesion, and the left neck.

**Figure 2:**
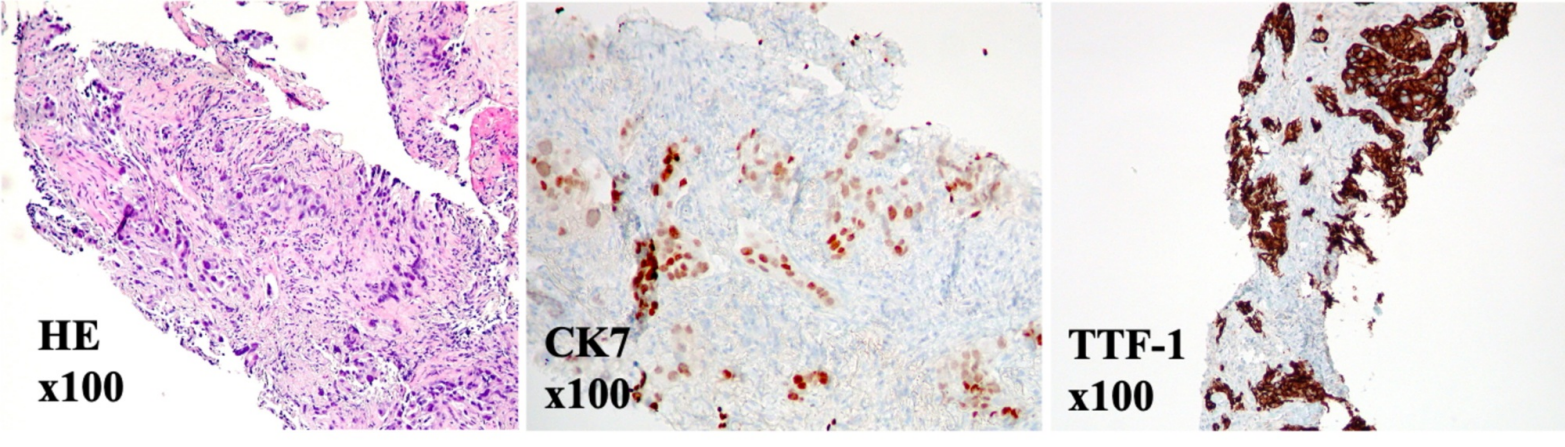
Endobronchial specimen pathology shows a poorly differentiated adenocarcinoma. Evaluation of the lung biopsy at medium power magnification reveals relatively large malignant cells with an invasive growth pattern tumor  [Haematoxylin and Eosin stain (HE) stain, x100]. Malignant cells are positive for cytokeratin 7 (CK7) and thyroid transcription factor-1 (TTF-1) immunostains. Malignant cells are negative for cytokeratin 5/6 and P63 immunostains (squamous cell differentiation markers; not pictured) and cytokeratin 20 (lower gastrointestinal tract marker; not pictured). Tumor morphology and this immunostaining profile are consistent with poorly differentiated adenocarcinoma of likely lung origin.

A 52-year-old female presented to the emergency department with an acute episode of shortness of breath. Her past medical history was notable for chronic tobacco smoking and a mixed chronic obstructive pulmonary disease-asthma phenotype. Subsequently, she underwent a workup that involved chest imaging, bronchoscopy with endobronchial biopsy, positron emission tomography/computed tomography (PET/CT) scans, and next-generation sequencing (Figures 1-2). Ultimately, she was diagnosed with EGFR/ALK/ROS1-negative, grade 3, stage IV NSCLC (T4N2M1b). PD-L1 was not over-expressed. She enrolled in an immunotherapy clinical trial with upfront nivolumab plus Ipilimumab therapy. Pre-enrollment transthoracic echocardiogram was completely unremarkable, and with a left ventricular ejection fraction estimated to be 69%. Six months later she had a follow-up PET/CT scan after two cycles of immunotherapy which showed partial response, but no evidence of disease progression. She continued to improve clinically while on combination immunotherapy, and had entirely negative PET-avid disease at each imaging interval. As a result, she remained on nivolumab and ipilimumab.

**Figure 3:**
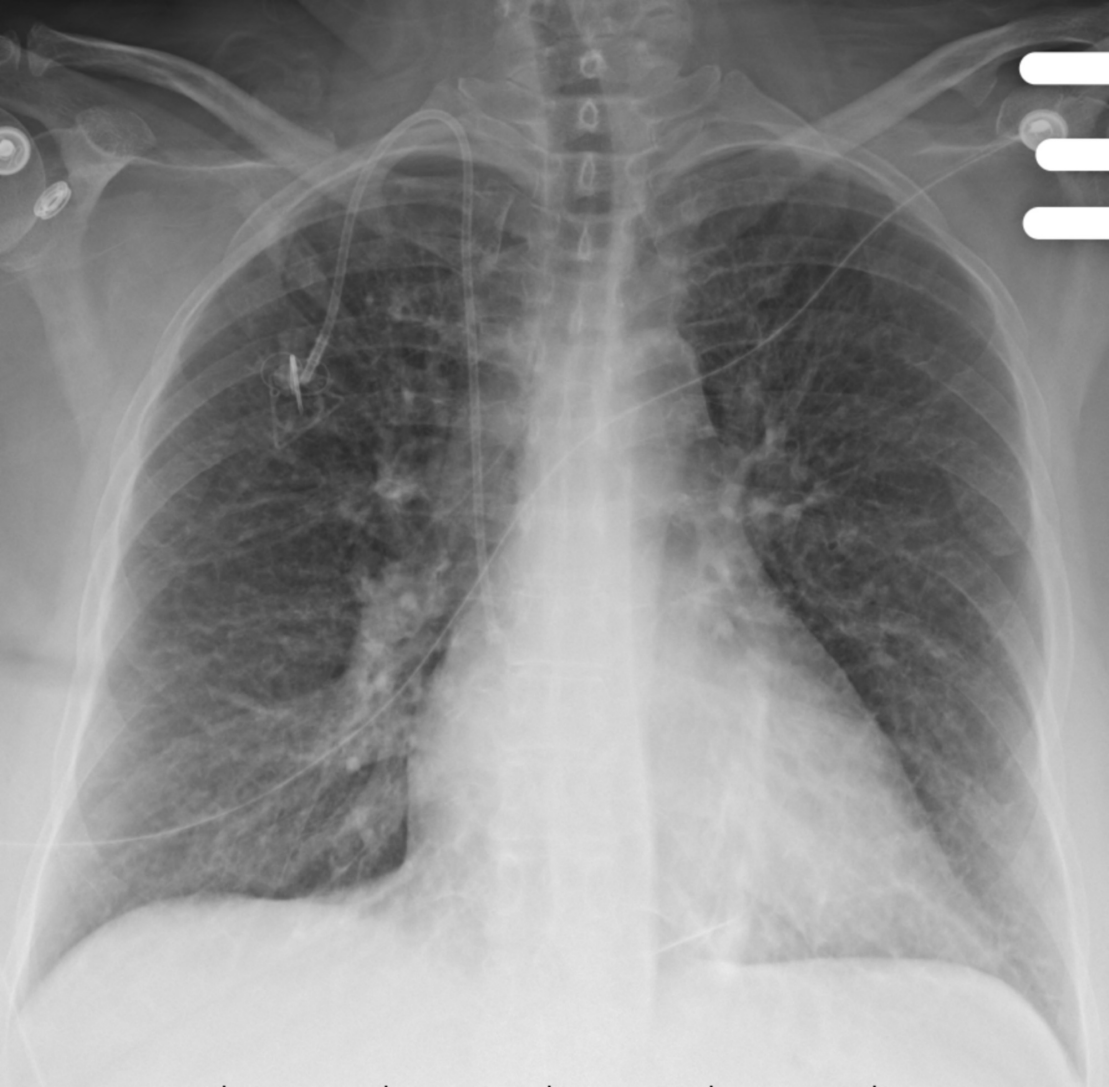
Chest X-ray shows bilateral interstitial markings. There are increased interstitial changes in the lungs bilaterally. Pulmonary vascularity is not congested. No pleural effusion is seen. Cardiac silhouette size is upper normal.

**Figure 4:**
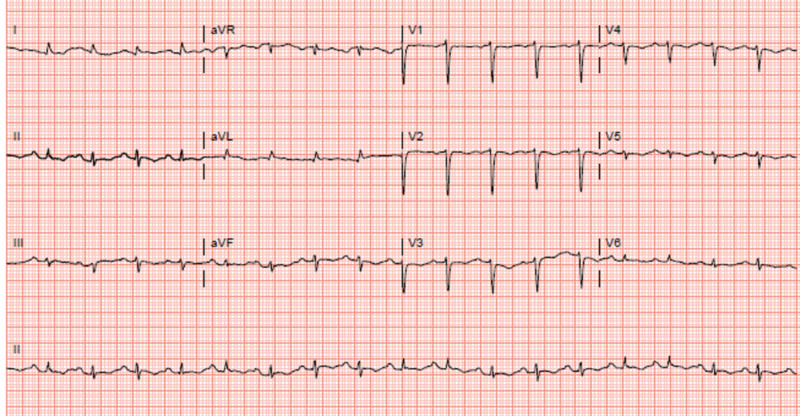
Electrocardiography obtained on admission for the chief complaints of acute shortness of breath and chest pain. Rate of 110 beats per minute. Intervals in milliseconds: PR 174, QRS duration 92, QT 342, QTc 464. Interpretation - Sinus tachycardia, low QRS voltage in extremity leads.

One year after being on combined immunotherapy, she presented to the emergency department with subacute dyspnea on exertion and anginal-like chest pain. Symptoms were associated with new-onset paroxysmal nocturnal dyspnea, 5-pillow orthopnea, and lower extremity edema. On initial evaluation, she was found to be hypoxic (peripheral oxygen saturation of 80% on room air), tachypneic (respiratory rate of 27), and borderline blood pressure of 94/67 mmHg. Her respiratory status was compromised to the point that she required noninvasive positive-pressure ventilation for her acute hypoxemic and hypercapnic respiratory failure. Chest x-ray findings suggested new interstitial edema (Figure 3). CT angiography found no filling defects to indicate pulmonary emboli. Also, no pericardial effusion was present, but cardiomegaly was noted. Electrocardiography was notable for mild sinus tachycardia and decreased amplitudes supporting low voltage (Figure 4). Initial serum laboratory testing was primarily equivocal. No obvious single etiologic agent was evident, such as an infectious, non-specific reactive inflammatory, autoimmune, malignant, or aseptic cardiopulmonary source (Table 1). Laboratory findings reinforced the importance of keeping a broad differential during the initial workup process. 

**Table 1:** Initial workup by serum laboratory testing is primarily equivocal. * No culture growth after 5 days of incubation. ** Clean urinalysis interpretation based on the absence of leukocyte esterase, nitrites, glucose, ketones, protein, blood, red/white blood cells, bacteria, and sediments. *** Significant elevation 5x above the upper limit of normal in cardiac biomarkers that have a remarkable doubling rate within the first six-hour interval after admission that eventually plateau.

Labs	Result	Reference range
White blood cells	13	4.8 – 10.8 10^3^/uL
Bands	0	0 – 8 %
Immature granulocytes	0.6	0 – 1.0 %
Neutrophils	49	51 – 75 %
Eosinophils	15	0 – 4 %
Procalcitonin	< 0.02	0 – 0.09 ng/mL
Epstein-Barr virus DNA	Negative	Negative
Adenovirus IgG	Negative	Negative
Enterovirus IgG	Negative	Negative
Culture*	No growth	No growth
Urinalysis**	Clean	Clean
Glucose	300	70 – 100 mg/dL
Hemoglobin A1c	6.6	4.1 – 5.6 %
CO2	17	22 – 32 mEq/L
Blood urea nitrogen	9	4 – 20 mg/dL
Creatinine	1.3	0.44 – 1.03 mg/dL
Glomerular filtration rate	45	> 60 mL/min
Anion gap	17	3 – 10 mEq/L
Alanine aminotransferase	57	14 – 54 U/L
Aspartate aminotransferase	46	15 – 41 U/L
Arterial blood gas		
pH	7.3	7.35 – 7.45
pCO2	38	35 – 45 mmHg
pO2	81	80 – 100 mmHg
Bicarbonate	19	22 – 26 mEq/L
Lactic acid	6	0.5 – 2.0 mEq/L
Creatine phosphokinase	190	38 – 234 U/L
Troponin I trend***	0.21, 2.30, 2.29	0 – 0.06 ng/mL
B-type natriuretic peptide	45	0 – 99 pg/mL
Cholesterol	220	0 – 199 mg/dL
LDL	140	0 – 130 mg/dL

She was admitted to the intensive care unit (ICU) for further evaluation of her concerning condition. Transthoracic echocardiogram was performed which revealed a severely reduced systolic function supported by an estimated left ventricular ejection fraction of 15-20%. Significant regional wall motion abnormalities were evident. Akinesis of the entire apical, septal, and lateral myocardium was observed. Pulmonary arteries systolic pressure was moderately increased and the inferior vena cava (IVC) was dilated. However, cavity size and wall thickness were normal and there were no valvular abnormalities. Left heart catheterization revealed normal coronaries with no blockages. Spirometry was performed at the bedside and showed a mixed ventilatory defect consistent with her chronic smoking history and mixed chronic obstructive pulmonary disease-asthma phenotype.

After an extensive and appropriate workup was performed, it was determined that she had new-onset heart failure, and likely myocarditis secondary to combination immunotherapy of a PD-1 inhibitor plus CTLA-4 inhibitor. This was based on her presentation, clinical status, acute deterioration in cardiac function, and her creatinine phosphokinase being in the upper range of normal. She was started on high dose intravenous (IV) steroids with methylprednisolone sodium succinate (125 mg) every six hours and underwent aggressive intravenous IV diuresis. She continued to be treated symptomatically in the ICU for her acute cardiomyopathy and likely immunotherapy-induced myocarditis. Cardiac biomarkers peaked and plateaued shortly after initiating steroids. Her oxygen requirements improved and she was weaned from non-invasive ventilatory support to low volumes of supplemental oxygen by nasal cannula shortly after starting steroids and IV diuretics. Follow-up transthoracic echocardiogram obtained on hospital day 8 showed near-complete resolution of her cardiac function. Systolic function was found to have improved to the lower limits of normal range with an estimated ejection fraction of 45-50%. In addition, no regional wall motion abnormalities, akinesis, or IVC dilation was noted. She continued to improve clinically and was deemed appropriate for hospital dismissal. She was dismissed on oral prednisone 60 mg with instructions to continue a slow steroid taper over a 6-week period.

She tolerated the outpatient setting well, and her cardiopulmonary symptoms continued to improve while on the slow steroid taper. At her 3-month follow-up visit, a monitoring transthoracic echocardiogram was obtained which showed continued cardiac function improvement with an ejection fraction of 50-55%. Also, at this time PET/CT was done which showed continued complete resolution of all her previous hypermetabolic activity of concern. This was consistent with a durable and long-lasting excellent response to one year of continual combination immunotherapy of a PD-1 inhibitor plus CTLA-4 inhibitor for her EGFR/ALK/ROS1-negative stage IV NSCLC. The patient remains alive, active, healthy, symptom-free, and in complete remission two and a half years after cessation of immunotherapy.

## Discussion

Immune checkpoint inhibitor therapy is being increasingly utilized and has become the standard of care in numerous cancers with promising results. However, irAEs not previously reported during clinical trials are emerging and can be life-threatening. Our patient received combination immunotherapy of nivolumab plus ipilimumab. Her low-expressed PD-L1 which lacked a driver mutation made such a combination highly favorable [8]. The distinct mechanisms of action of these two checkpoint inhibitors have also provided the rationale for using them in other types of cancers like melanoma and advanced renal cell carcinoma, among others [9, 10].

Monotherapy with either nivolumab or ipilimumab has been associated with severe and fatal immune-related adverse events [11, 12]. However, the combination of nivolumab plus ipilimumab is associated with increased toxicity relative to single-agent immunotherapy [13]. CheckMate 067 trial extensively studied the adverse events of combining nivolumab and ipilimumab. The study found that the incidence of grade 3 or 4 toxicity with the combination, after a minimum follow-up of five years, was increased compared with either single agent (59% versus 23% and 28%, respectively, for nivolumab and ipilimumab) with two treatment-related deaths reported with the combination, and one each of the nivolumab and ipilimumab treatment arms [9]. No unique toxicities were attributed to the combination therapy that were not previously seen with either agent alone. Such toxicities were managed similarly to those arising from either treatment with monotherapy.

The most common immune-related toxicities include dermatitis, endocrinopathies, colitis, hepatitis, and pneumonitis. Myocarditis, recognized as an uncommon adverse reaction, has also recently been reported in few cases in cancer patients treated with these agents and it may result in poor outcomes if not properly recognized and managed. While the precise mechanism of action remains to be elucidated, the general consensus pertains to the dysregulation of the auto-reactivity mechanisms that are usually maintained by immune checkpoints [4]. However, and for myocarditis specifically, prior reports have described a potential role for PD-1 in cardiomyocyte protection against autoimmune attacks as demonstrated in PD-1 deficient murine models that developed dilated cardiomyopathy [14].

In pharmacovigilance studies, the incidence of myocarditis was higher in patients treated with the combination of nivolumab plus ipilimumab compared with nivolumab alone (0.27% versus 0.06%) [6]. As in our patient, autoimmune myocarditis presents with nonspecific symptoms of respiratory distress and a wide range of symptoms of cardiac dysfunction and can occur in patients with no previous cardiac disease. The time from starting checkpoint inhibitors to exhibiting these complications is variable with an average of 4-8 weeks [15]. However, fatal myocarditis, with autopsy findings, has been reported after a single treatment with the combination of nivolumab plus ipilimumab [16].

Steroids are used to treat immunotherapy-related myocarditis. As per the American Society of Clinical Oncology’s general approach to toxicity management multidisciplinary panel and the Society for Immunotherapy of Cancer's recommendations, subtle cases (grade 2 or moderate) are simply treated by withholding immunotherapy [17, 18]. Prednisone 0.5 mg/kg/day (or equivalent) is to be started if symptoms do not resolve within a week of withholding immunotherapy. Should symptoms or toxicity be grade 1 or less, immunotherapy can be resumed. Severe or life-threatening (grade 3 or 4) cases are treated with high doses of corticosteroids (prednisone 1-2 mg/kg/day or equivalent) with gradual tapering for at least a month when symptoms subside to grade 1 or less plus permanently discontinuing immunotherapy, as in our patient. In patients without an immediate response to high-dose steroids, the early institution of cardiac transplant rejection doses of steroids (methylprednisolone 1 g every day) and the addition of a steroid-sparing agent (eg, mycophenolate, infliximab, or anti-thymocyte globulin) should be considered [19].

When retreatment with immunotherapy is required after prior toxicity, data are limited on the specific patient populations who should not be offered retreatment, and clinical judgement is necessary. Retreatment is generally discouraged in patients who received steroid doses equivalent to prednisone 10 mg daily or higher for treatment of the initial episode of myocarditis, as concurrent use of Prednisone is associated with reduced efficacy of immunotherapy [20]. Basically, those who survived a frequently fatal immune-related cardiotoxicity are not routinely offered retreatment. For these patients, the optimal choice of retreatment agent varies in clinical practice, although patients who experience severe toxicity from initial CTLA-4 blockade are typically offered retreatment with single-agent PD-1 or PD-L1 monotherapy rather than repeat CTLA-4 blockade. Dose reductions of immunotherapy are not recommended with retreatment, as this approach has not been assessed in clinical trials. Our patient had a complete or sustained response to the initial regimen and did not require retreatment or further intervention.

This case highlights the importance of pre-treatment cardiac screening with basic investigations such as electrocardiogram, cardiac and inflammatory markers despite the rarity of this condition due to its potentially fatal complications. It also signifies the multidisciplinary team approach involving early cardiology input to manage the cardiac complications, particularly cardiac arrhythmias and left ventricular systolic dysfunction.

## Conclusions

Immunotherapy-related myocarditis appears to be an idiosyncratic reaction and likely not dose dependent. No predictive biomarker is currently available for this rare toxicity, and no effective preventive methods are established. Diagnosis is challenging due to the nonspecific presentation with a very wide range of differential diagnoses. Therefore, it is imperative to have a high index of suspicion as most of these agents are either recently approved or mainly handled by oncologists in the outpatient setting. Management is conducted on a case by case basis and generally involves an extensive workup for the more common causes of various diseases before attributing the cause to immunotherapy. Recommendations with high quality evidence are lacking and the approach for treatment is based on the subjective reporting of the grade severity of an adverse event. Data is limited for the benefits of retreatment after prior toxicity and careful risk-benefit discussion with patients who are candidates for retreatment is crucial.
